# Editorial: Adoption of artificial intelligence in human and clinical genomics: volume II

**DOI:** 10.3389/fmolb.2026.1949944

**Published:** 2026-08-17

**Authors:** Li Zhang, Matteo Becatti

**Affiliations:** 1 Department of Computer Science, Royal Holloway, University of London, London, United Kingdom; 2 Department of Experimental and Clinical Biomedical Sciences “Mario Serio”, University of Firenze, Firenze, Italy

**Keywords:** artificial intelligence (AI), clinical genomics, explainable AI, machine learning, precision medicine

Artificial intelligence (AI) and machine learning are rapidly transforming human and clinical genomics, offering new opportunities for the interpretation of complex biological data, the identification of disease biomarkers, the improvement of diagnostic workflows, and the development of precision medicine strategies. As genomic technologies continue to generate large-scale and multidimensional datasets, AI-based approaches are becoming increasingly important for extracting clinically meaningful information and translating genomic discoveries into healthcare applications.

The articles included in the Research Topic “*Adoption of Artificial Intelligence in Human and Clinical Genomics: Volume II*” collectively illustrate the expanding role of AI across different areas of genomics, including biomarker discovery, variant interpretation, drug development, precision oncology, and clinical natural language processing. This article Research Topic comprises three Original Research articles and two Review articles, providing both novel methodological advances and comprehensive overviews of emerging trends that are shaping the future of AI-driven genomics and precision medicine.

Huang et al. applied an integrated bioinformatics and machine learning approach to diabetic kidney disease with concurrent vascular calcification, a clinically important condition associated with increased cardiovascular risk (Huang et al.). By combining differential expression analysis, weighted gene co-expression network analysis, and multiple machine learning algorithms, the authors identified key candidate genes and developed a diagnostic model. Their findings suggest that AI-supported analysis of transcriptomic data can help uncover molecular signatures relevant to early diagnosis, disease stratification, and future therapeutic targeting.

The potential of AI in therapeutic discovery is further demonstrated by the Original Research article of Lee et al., who developed DeepKinome, a deep learning-based regression model for the quantitative prediction of kinase-compound binding affinity (Lee et al.). By moving beyond binary classification and predicting continuous binding affinity values, this study provides a more refined approach to modeling kinase inhibition. Importantly, the use of explainable AI allowed the identification of biologically meaningful amino acid regions contributing to model predictions. This work illustrates how deep learning can support drug discovery by improving the prediction of molecular interactions while also offering insights into their biological basis.

The Research Topic also emphasizes the role of AI in precision oncology. In a Review article, Zehra et al. examined the integration of AI and machine learning approaches with microRNA analysis for cancer diagnosis, prognosis, and personalized treatment (Zehra et al.). MicroRNAs represent promising non-invasive biomarkers because of their stability in body fluids and their cancer-specific expression patterns. The review highlights how AI-based models can identify miRNA signatures associated with early cancer detection, disease progression, therapeutic response, and chemoresistance. At the same time, the authors underline important limitations, including data heterogeneity, lack of standardization, demographic bias, and the need for external validation before clinical implementation.

Another major challenge in genomic medicine is the interpretation of noncoding variants, which represent a large proportion of disease-associated genetic variation. In a Review article, Wang et al. provided an overview of evolving computational paradigms for noncoding variant pathogenicity prediction (Wang et al.). The authors described the transition from traditional annotation-based methods to advanced machine learning, deep learning, and genome language model-inspired approaches. These methods are increasingly able to integrate genomic, epigenomic, regulatory, and structural information to improve the prioritization of clinically relevant variants. This contribution highlights the importance of AI in addressing one of the most complex tasks in modern genomics: understanding the functional and pathogenic consequences of variation outside protein-coding regions.

Beyond sequence-based analysis, AI is also transforming the management of clinical genomic information contained in unstructured medical texts. Sultangaziyeva et al. presented GENEXOM, the first multi-layer annotated corpus of Russian-language clinical exome sequencing reports (Sultangaziyeva et al.). The corpus includes annotations for genetic, phenotypic, and clinical entities and relations, aligned with international standards guidelines. By applying transformer-based models for named entity recognition and relation extraction, the authors demonstrated the potential of clinical natural language processing to support automated variant interpretation, phenotype-disease mapping, and biomedical knowledge graph construction.

Taken together, the studies included in this Research Topic demonstrate the breadth and maturity of AI applications in human and clinical genomics. They show how machine learning and deep learning can contribute to biomarker identification, disease diagnosis, drug discovery, cancer risk prediction, variant classification, and clinical information extraction. Importantly, these contributions also reflect a broader transition from descriptive genomic analysis toward predictive, interpretable, and clinically actionable genomic medicine. Despite these advances, several challenges remain. The successful adoption of AI in clinical genomics requires transparent and explainable models, rigorous validation across independent and diverse datasets, harmonized data standards, and careful assessment of algorithmic bias. In addition, the integration of multimodal data, including genomic, transcriptomic, epigenomic, clinical, imaging, and environmental information, will be essential to fully realize the potential of AI-driven precision medicine.

In conclusion, this Research Topic highlights the growing convergence between artificial intelligence and genomics as a key driver of future biomedical innovation. The contributions presented here demonstrate that AI is no longer only a computational support tool, but an increasingly central component of genomic research and clinical translation. Continued collaboration among computational scientists, geneticists, clinicians, bioinformaticians, and data scientists will be crucial to ensure that AI-based genomic technologies are reliable, equitable, interpretable, and ultimately beneficial for patient care.

